# The re-identification risk of Canadians from longitudinal demographics

**DOI:** 10.1186/1472-6947-11-46

**Published:** 2011-06-22

**Authors:** Khaled El Emam, David Buckeridge, Robyn Tamblyn, Angelica Neisa, Elizabeth Jonker, Aman Verma

**Affiliations:** 1 Children's Hospital of Eastern Ontario Research Institute, 401 Smyth Road, Ottawa, Ontario K1J 8L1, Canada; 2 Pediatrics, Faculty of Medicine, University of Ottawa, Ottawa, Ontario, Canada; 3 Department of Epidemiology, Biostatistics and Occupational Health, McGill University, 1140 Pine Avenue West, Montreal, Quebec H3A 1A3, Canada

## Abstract

**Background:**

The public is less willing to allow their personal health information to be disclosed for research purposes if they do not trust researchers and how researchers manage their data. However, the public is more comfortable with their data being used for research if the risk of re-identification is low. There are few studies on the risk of re-identification of Canadians from their basic demographics, and no studies on their risk from their longitudinal data. Our objective was to estimate the risk of re-identification from the basic cross-sectional and longitudinal demographics of Canadians.

**Methods:**

Uniqueness is a common measure of re-identification risk. Demographic data on a 25% random sample of the population of Montreal were analyzed to estimate population uniqueness on postal code, date of birth, and gender as well as their generalizations, for periods ranging from 1 year to 11 years.

**Results:**

Almost 98% of the population was unique on full postal code, date of birth and gender: these three variables are effectively a unique identifier for Montrealers. Uniqueness increased for longitudinal data. Considerable generalization was required to reach acceptably low uniqueness levels, especially for longitudinal data. Detailed guidelines and disclosure policies on how to ensure that the re-identification risk is low are provided.

**Conclusions:**

A large percentage of Montreal residents are unique on basic demographics. For non-longitudinal data sets, the three character postal code, gender, and month/year of birth represent sufficiently low re-identification risk. Data custodians need to generalize their demographic information further for longitudinal data sets.

## 1 Background

There are increasing pressures to make individual-level data more readily available for research and policy making [1-5], and there are believed to be many benefits to doing so [2,6-19]. Such broad disclosures of health data also pose significant privacy risks [20]. Moreover, the public is uncomfortable providing personal information, or allowing their personal information to be used for research, if they do not trust the organization collecting and using the data. Individuals often cite privacy and confidentiality concerns and lack of trust in researchers as reasons for not having their health information used for research [21]. One study found that the greatest predictor of patients' willingness to share information with researchers was the level of trust they placed in the researchers themselves [22]. A number of US studies have shown that attitudes toward privacy and confidentiality of the census are predictive of people's participation [23,24], and also that there is a positive association between belief in the confidentiality of census records and the level of trust one has in the government [25]. These trust effects are amplified when the information collected is of a sensitive nature [25,26].

One way to retain the trust of the public is to ensure that the risk of re-identification is low before the disclosure of data to researchers or at the earliest opportunity after collection [21,27-35]. As many as 86% of respondents in one study were comfortable with the creation and use of a health database for research purposes where individuals could not be re-identified, whereas only 35% were comfortable with such a database that included identifiable information [33]. Furthermore, many research ethics boards (REBs) will waive the consent requirement if the first "use" of the data is to de-identify it [36,37].

It is generally accepted that individuals can be easily re-identified through their basic demographics because these demographics make individuals unique in the population. The uniqueness of individuals is often used as a surrogate measure of re-identification risk [38-43]. In the US it has been estimated that between 63% and 87% of the population is unique on their basic demographics [39,41], and more than 99% of Dutch citizens are [43]. Demographic information, such as residence postal code, date of birth, and gender, is included in many health data sets disclosed for secondary purposes, such as research.

There has been a dearth of studies on the uniqueness of Canadians on their basic demographics, and no studies have been performed on the re-identification risk from longitudinal data sets in any jurisdiction. Longitudinal data sets will often include an individual's location trail over time. For example, in an electronic medical record (EMR) the residence of the patient is often updated at every visit; therefore an EMR data set will have a patient's residence trail.

The purpose of this study is to evaluate the uniqueness of Montrealers using longitudinal demographic information over an 11 year period. Based on this analysis we provide guidelines and a list of disclosure policies that will ensure that the risk of re-identification is acceptably low.

### 1.1 Definitions

We define a quasi-identifier as a variable in the data set that can be used to probabilistically identify an individual. General examples of quasi-identifiers include sex, geographic indicators (such as postal codes, census geography, information about proximity to known or unique landmarks), and event dates (such as birth, admission, discharge, procedure, death, specimen collection, visit/encounter).

Uniqueness means that there is only one individual with those values on the quasi-identifiers in the population. For example, if our quasi-identifiers are age, gender, and postal code, and there is only one 90 year old female in the postal code "H3A 2T5", then she is a unique individual on these quasi-identifiers. Other variables that are not considered quasi-identifiers are not taken into account in the evaluation of uniqueness.

### 1.2 Methods of Attack

In this section we illustrate why population uniqueness makes it easy to re-identify individuals in a disclosed data set. We assume that the data is being disclosed for research purposes.

**Figure 1:**
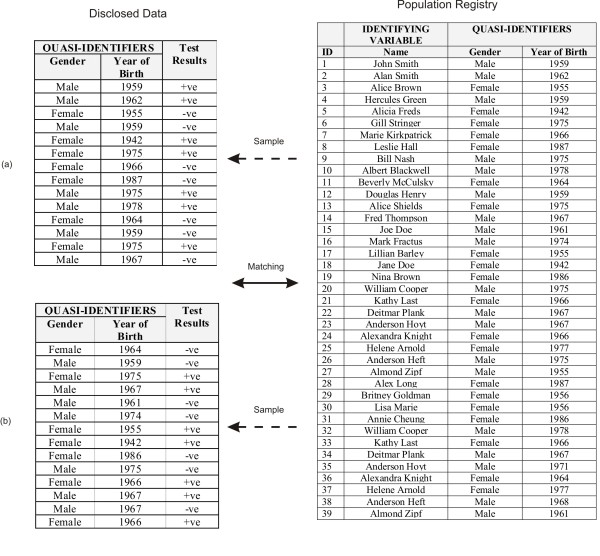
**Example of data sets matched against a population registry with uniques**.

Consider the scenario illustrated in Figure 1. We have a population registry, for example, the census, which holds the names and some basic demographics of all individuals. Any particular data set that is disclosed for research purposes will be a subset of that population registry. The figure shows two disclosed data sets, (a) and (b), which contain the basic demographics as well as sensitive test results.

If an adversary has background information about Alan Smith, such as his gender and year of birth, and knew that Alan's record was in the disclosed data set (a), then it would be relatively easy for the adversary to find Alan's record. The reason is that Alan Smith is unique in the population: there is only one male born in 1962 in the population. If an individual is unique in the population then that individual will also be unique in a sample. Therefore, when the adversary finds the <male,1962 > record in data set (a) it will be Alan with certainty. An adversary would know Alan's basic demographics because the adversary may be Alan's neighbour, co-worker, spouse or ex-spouse, or maybe Alan is a famous person and his demographics are publicly known.

Now consider the disclosed data set (b), which is another sample from the population. In this example the adversary has some background demographic information about Alice Shields. However, the adversary does not know if Alice is in the disclosed data set or not. For example, the data set may be a sample survey and an adversary would not know who was asked to participate, and out of those, who responded. However, Alice is unique in the population: there is only one female born in 1975 in the population. If the adversary finds a record in the disclosed data set with a female born in 1975 then the adversary will know with certainty it is Alice, and therefore Alice's record would be re-identified.

The above examples illustrate that population uniqueness makes disclosed data vulnerable to re-identification by an adversary who has basic demographics about targeted individuals. This is true whether the adversary knows a priori if the target individuals are in the disclosed data set or not.

Consider another scenario, that the adversary had access to a population registry, such as a voter registration list, which would contain the basic demographics [44]. The adversary was going to match the disclosed data set (a) against the voter list for re-identification purposes. This is different from the above two scenarios because now the adversary is not trying to re-identify a specific targeted individual, but rather trying to re-identify any record that matches. The record in the disclosed database with a male born in 1962 will match with record number 2 in the voter registration list. Because this is Alan's record and Alan is unique in the population, this will be a correct match with certainty. The more unique individuals in the population, the greater the number of correct matches under this method of attack.

It is important to make a distinction between population uniqueness and sample uniqueness. Consider the record <male, 1959 > in table (b) in Figure 1. This record is unique in the sample but is not unique in the population. If an adversary was trying to re-identify the record for John Smith in table (b) and did not know if John Smith was in table (b), then it would not be possible to determine with certainty whether that matching record in table (b) really belongs to John Smith or not. Therefore, it is population uniqueness that is relevant as a measure of re-identification risk, and not sample uniqueness.

### 1.3 The Identifiability of Demographics

While Canadian privacy laws apply to identifiable personal information [45], not many of them provide an explicit definition of what is meant by "identifiable information". For example, in Ontario's Personal Health Information Protection Act identifying information is defined as "information that identifies an individual or for which it is reasonably foreseeable in the circumstances that it could be utilized, either alone or with other information, to identify an individual" and Alberta's Health Information Act defines individually identifying information where "the identity of the individual who is the subject of the information can be readily ascertained from the information". Statutory tests for identifiability range from lower thresholds of what is "reasonably foreseeable" or can be "reasonably expected" to identify individuals, to higher thresholds of what is "readily ascertainable" or "obvious". None of these thresholds expressly require application from any particular perspective (for example, a highly sophisticated expert, or conversely, a lay member of the general public) nor do they make express reference to the level of resources, time or effort necessary to re-identify individuals. Furthermore, they do not define a metric to measure risk on, nor a quantitative value for what would be acceptable risk.

In our context, the implication is that basic demographics, such as date of birth and postal code information, may in some cases be considered identifiable information and in other cases may not be, depending on which subjective test is applied. In practice, this results in considerable variability, and calls for more precise and quantitative approaches to measure identifiability and developing acceptable risk thresholds in a Canadian context.

There are some similarities to the US, where the Health Insurance Portability and Accountability Act (HIPAA) Privacy Rule defines two standards for de-identification. The Safe Harbor standard specifies 18 variables that must be removed, including date of birth and full ZIP codes. While very precise, it could result in significant information loss [46]. The Statistical standard requires an expert to certify that the "the risk is very small that the information could be used, alone or in combination with other reasonably available information, by an anticipated recipient to identify an individual who is a subject of the information". This is similar to the Canadian approach in that it is possible to allow more detailed information to be disclosed if the risk of re-identification can shown to be very small. This necessitates the definition of re-identification risk metrics and what "very small" means.

## 2 Methods

### 2.1 Data Set

The provincial health insurance claims database of Quebec holds demographic information on all citizens that have health insurance. Because this is publicly financed insurance, it effectively captures the whole population.

We sampled records on 928,708 individuals living in the Montreal Census Metropolitan Area, as defined in the 2006 Census, from the provincial health insurance database. A random sample of 2.5% of individuals was taken each year over an 11 year period between 1996 and 2006 inclusive. Because by chance some individuals appeared more than once, this data set represents approximately 25% of the population of Montreal.

For each individual we obtained their date of birth, gender, date of death if they died during that period, as well as their full postal code. Individuals had their full residence postal codes determined at the beginning of each year by the provincial insurer. We therefore had postal code information for each sampled individual for each of the 11 years (with the exception of births and deaths, as noted below).

The Quebec provincial health insurer updates the population addresses in its database every week using three sources: (a) a government services web site where the public can change their address directly and this affects their records in multiple departments, such as elections, social insurance, provincial taxes, and health insurance, (b) the automobile insurance service (la Societe de l'assurance automobile du Quebec) where drivers must renew their license every year and update their address, and (c) renewals by the public of their health insurance cards, which must happen every four years.

Despite these processes to ensure correct information, one percent of the patients had to be removed because they had no postal codes (i.e., no postal codes at all for any year), and 5% were removed because they had inconsistent postal codes (e.g., postal codes before birth and postal codes after death).

### 2.2 Births and Deaths

For patients who were born during that 11 year period or died during that period, specific unborn ("UUU-UUU") and dead ("DDD-DDD") postal codes were used for the empty years. By doing so, we assume that an adversary would know that an individual was not born or is dead and therefore can use that background knowledge in a re-identification attack. For example, assume that there were two males born in 1940 who were now dead. One had a missing postal code and one with a "DDD-DDD" postal code. If an adversary was trying to re-identify the record of a deceased John Smith who was born in 1940, then the adversary would know which record belongs to John Smith. If we did not account for death then that adversary knowledge would not help the adversary. If both records had missing postal codes then knowledge that John Smith was deceased would not help the adversary determine which was the correct record. The missing values may be due to a clerical error or because both individuals died or some combination of these two explanations. For such an adversary the uniqueness values will be lower than the values we present here, meaning that our uniqueness estimates would be lower for an adversary who does not have birth/death background information.

By taking the first option, and indicating births and deaths, we assume a more knowledgeable adversary.

The data set we analyzed consisted of 3,530 unique postal codes.

### 2.3 Measuring Risk

We used uniqueness as the measure of re-identification risk. This is a commonly used metric of re-identification risk in the disclosure control literature [38-43].

The three quasi-identifiers we used were date of birth, postal code, and gender. For example, if we consider the full date of birth, the full postal code, and gender, then the uniqueness measure gives us the proportion of individuals in Montreal who are unique on their values on these three variables. Since we only have a sample, population uniqueness was estimated using the Zayatz estimator [47].

### 2.4 Analysis

In this analysis, we will refer to a series of postal codes over time as a 'residential trail'. For example, the postal code information for an individual over three years is a three year residential trail.

Each study point consists of a set of generalizations on the three variables and the number of years of the residential trail. A uniqueness value was computed for each study point.

The date of birth was generalized to month and year of birth, then year of birth. The full 6-character postal code was generalized to 5 characters, 4 characters, and so on until 1 character. The postal code generalization was performed by cropping the last character. For example, a 6 character postal code "H3A 2T5" would be cropped to a 5 character postal code as "H3A 2T", and so on.

The analysis was performed for residential trails of one year, 2 years, and so on in 1 year increments to 11 years. For the one year residential trails we selected a year at random out of the 11 with equal probability and computed uniqueness for that. Initially we repeated this 1000 times, each time selecting a year at random, with the average uniqueness computed across the 1000 runs. For two years and more, we randomly selected years that were not necessarily contiguous. We used a change threshold of 0.0001 for average uniqueness, which would allow us to stop the iterations sooner if the amount of change from one iteration to the next over 10 iterations is smaller than the threshold. In practice then, the average uniqueness was computed across 100 to 500 iterations before the stopping criterion was met.

We also examined uniqueness for youth and adults separately. An individual was considered to be a youth if s/he was less than 20 years old during the sampled period. For example, if we are looking at a two year residence trail, then for either of the sampled 2 years an individual would have to be less than 20 years to be considered a youth.

### 2.5 Ethics

The analysis of this data set was approved by the Research Ethics Board (REB) of the Children's Hospital of Eastern Ontario Research Institute, the McGill University REB, the Quebec provincial health insurance agency (RAMQ), and the privacy commissioner of Quebec.

## 3 Results

Approximately 48% of the population did not change their residential address at any time during the 11 year period, 21% changed address once, 17% twice, 8% three times, and 6% four times or more.

**Figure 2:**
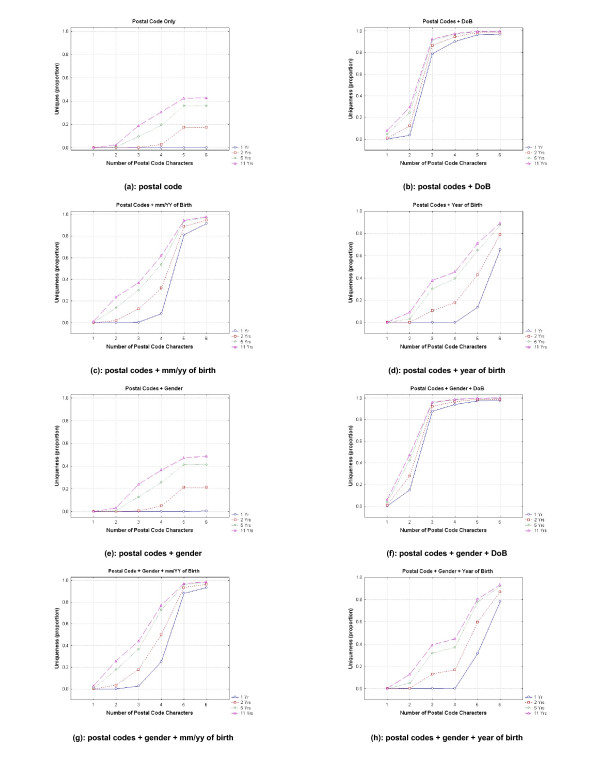
**Estimated uniqueness results for different levels of generalization on the three demographic variables for 1 year, 2 years, 5 years, and 11 years**.

The estimated uniqueness results for 1 year, 2 years, 5 years, and 11 years are shown in Figure 2. Just under 30% of the sample was youth, and the remainder were adults. The results for adults and youth separately are not presented in detail here because they are very similar to the results for the full data set. However, they are included in their entirety in additional file 1.

Uniqueness tended to be slightly higher for adults compared to youth. This is not unexpected as there are many possible values for adult dates of birth compared to youth, resulting in many more 'bins' that a birth date may fall into, and therefore increasing the chances of unique values.

The first interesting observation is for a single year residential trail: the proportion of individuals is close to zero for the full six character postal code (see Figure 2 panel a). Using only the postal codes, as the length of the residential trail increases, the proportion of individuals who are unique grows. Over two years that proportion grows to 17.4% of the population, over 5 years 35% of the population are unique, and approximately 43% are unique on their residential trail over the 11 years. A significant proportion of the population has a unique residence pattern even over short periods of time, as small as two years.

When the full date of birth is used together with the full postal code, then approximately 98% of the population are unique with only one year of data (panel b). When the full date of birth and a multi-year residential trail are considered, then almost all of the population is unique. This means that date of birth and full postal code, together, are a unique identifier for Montrealers. If we add gender (panel f) then the whole population is unique. Reducing the granularity of the postal code to 1 character together with the full date of birth does reduce the proportion of uniques considerably, but even then it still has a nontrivial magnitude at almost 5% uniqueness for a five year residential trail.

Using a one year residential trail with postal code and gender together does not make a high proportion of the population unique (panel e - almost zero percent uniqueness), but when 2 years or more of data are used, the percentage of unique increases beyond 20%.

The differences in uniqueness are most pronounced when we consider shorter residential trails than for longer periods. For example, the difference in uniqueness between a single year and a 2 year residential trail is larger than the difference between a 10 year and an 11 year trail. This means that from a re-identification risk perspective, reducing a longitudinal data set from 11 years to say 10 years may not have much of an impact, whereas reducing it from 3 years to 2 years the drop in risk could be significant.

In Canada, it is common practice to disclose health information with only three characters of the postal code under the general belief that this results in low re-identification risk. For a single year data set, the three character postal code, gender, and month/year of birth represents quite a low uniqueness value (panel g) and therefore would be considered to have low re-identification risk. If the three character postal code is combined with the full date of birth (panel b), however, close to 80% of the population is unique. Therefore, whether a three character postal code represents acceptable re-identification risk or not will depend on what are the other quasi-identifiers that are disclosed: it is not possible to make a meaningful risk assessment by considering only the postal code if there are other quasi-identifiers in the data set.

If one is considering the disclosure of longitudinal data, what was acceptable for a single year may not be acceptable any more. For example, a five year residential trail of the three character postal code, gender, and month/year of birth make close to 40% of the population unique (panel g), which is quite different from the cross-sectional risk. Consequently, one must determine the length of the residential trail to make accurate re-identification risk assessments.

## 4 Discussion

### 4.1 Summary

We performed an analysis of the uniqueness of Montreal residents on basic demographics. The results indicate that on basic demographics, the population uniqueness is quite high. The inclusion of residence postal code information over time (the residence trails) ensures that a large percentage of the population is unique, even if we remove gender and generalize the date of birth. To properly assess the risks one must consider all of the quasi-identifiers and the length of the residential trail.

### 4.2 Practical Implications

Our results can be used by data custodians and research ethics boards to decide if the risk of re-identification is sufficiently low for the disclosure of health information for research, and possibly other secondary purposes. Thus far such decisions have been based more on intuition and less on actual evidence.

While there are no globally accepted standards for deciding when the value of uniqueness is too high, there are some precedents that one can consider.

There have been attempts at empirically measuring the actual re-identification risk of HIPAA Privacy Rule Safe Harbor data sets. One analysis concluded that 0.04% of the population is unique for a Safe Harbor compliant set of variables [48,49]. Another study evaluated the proportion of records that can be re-identified in a Safe Harbor compliant sample and found that only 0.01% can be correctly re-identified [50]. Recent work has shown that the proportion of unique individuals implied by Safe Harbor depends on the state one is referring to [44]: the re-identification risk varies from 0.25% of the population being unique in Wyoming to less than 0.01% for California (at least a 25 times difference in re-identification risk). However, it has been argued that the Safe Harbor standard is most useful if one is making data publicly available, and may be too stringent for research contexts [46].

Previous disclosures for research purposes, for example of cancer registry data, have deemed thresholds of 5% and 20% population uniqueness as acceptable thresholds to use for research data [51-53]. The higher threshold would be suitable where the recipient is more trusted (for example, internal or affiliated researchers) and the lower one for external researchers with no existing relationship with the data custodian. With these guidelines, a custodian can use our results to decide how much generalization to apply to a data set, and if it is a longitudinal data set, how many years of data to disclose before the risk becomes unacceptably high.

In additional file 2 we provide all of the possible values on the demographics that would be acceptable under the 5% and 20% thresholds. These represent disclosure policies that can be justified, assuming the two thresholds. For example, in the cross-sectional case (1 year) a data set with the full date of birth, gender, and the first character of the postal code would be acceptable under both the 5% and 20% thresholds. However, if the postal code had 3 characters then this would not be acceptable. This should provide data custodians and data users with concrete guidance for data disclosures.

It is also important to note some caveats to the practical application of our results by highlighting two assumptions: (a) a disclosed data set is a simple random sample from the population, and (b) the adversary does not know who is in the disclosed data set.

When we consider our methods of attack discussed earlier, the proportion of the population that is unique is a good reflection of re-identification risk if the disclosed data is sampled with equal probability from the population (of Montreal). This is because the expected proportion of unique records in a sample will be the same as the proportion of unique records in the population [54]. However, if the disclosed data set departs markedly from a simple random sample, then the uniqueness may be quite different. For example, if a data set consists of only females aged 13 to 17, then the number of possible equivalence classes on the demographic quasi-identifiers and over time will be much smaller than for all youth. This particular subset will likely have a different proportion of individuals that are unique compared to the whole population. While the simplifying assumption of simple random sampling is often used in the disclosure control literature, one should be cognizant of its limitations.

We also make the assumption that the adversary does not know who is in the disclosed data set. If the adversary knows who is in the data set then population parameters do not matter in the evaluation of re-identification risk [55]. For example, the year of birth and 4 character postal code for youth makes 9.2% of the population unique. If we have a (sample) data set of 1000 records, it could be that, say, 50% of the records in that data set are unique on the year of birth and the 4 character postal code. If an adversary knew that Alice was in that disclosed data set then the risk for Alice would be much higher than 9.2%. A detailed review of the conditions for deciding whether it is likely that an adversary would know if an individual is in a disclosed data set or not was provided in a recent article [55]. For instance, if a research study is sampling patients at random from an insurance claims database then it would not be possible for an adversary to know who is in that sample. On the other hand, if teenage participants in a drug use survey need consent from their parents to respond, then the parents will know if their children are in the data set. Therefore, if it is plausible for an adversary to know who is in the disclosed data set then sample uniqueness is the relevant re-identification risk metric rather than population uniqueness.

### 4.3 Sensitivity Analysis

Six percent of the records were removed in our analysis. We found that 1% of the records did not have a postal code and were removed. Had we kept these records in the data set as null records that could have artificially reduced our uniqueness estimates. Also, 5% had inconsistent records - since we did not know the cause of the inconsistency (the date of birth/death or the postal code), these records were also removed. The most likely reason for these inconsistencies is a data entry error. There is no a priori reason to believe that these data entry errors were systematic in that they would be more likely in unique versus non-unique individuals.

If we assume that the 6% of the records that we removed were a random sample from the full data set, then there would be no impact on our uniqueness values. If all of the records that were removed were unique, our uniqueness values would increase slightly had they somehow been corrected and retained. If none of the records that we removed were unique, our uniqueness values would decrease slightly had they somehow been corrected and retained.

**Figure 3:**
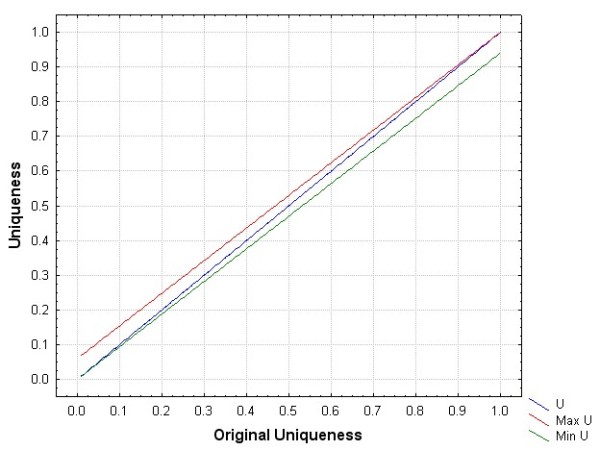
**The maximum and minimum uniqueness values had we retained the records with data entry errors**.

The changes in the uniqueness value are shown in Figure 3. This graph shows how the uniqueness estimates for a data set would be inflated/deflated under the two extreme conditions described above. At low uniqueness values, there would be the biggest increase if we assumed that the removed records were all unique and retained them. At high uniqueness values, there would be the biggest decrease if we assumed that none of the removed records were unique and we removed them.

In terms of impact on the acceptable values on the three quasi-identifiers in additional file 2, we are mostly concerned with the case where uniqueness is low. Where our results show a uniqueness of 5%, the actual uniqueness may be slightly higher at 10% under the extreme situation that all the records we removed were also unique, and at 20% the actual uniqueness may be as high as 25%. However, in practice it is highly unlikely that all records with data entry errors are unique.

### 4.4 Representativeness of the Results

In this section we examine the extent to which the results can be generalized beyond Montreal and how they compare to the results in the US.

#### 4.4.1 Generalization to Other Urban Canadian Centers

One important question is whether the results obtained from Montreal can be generalized to other urban areas in Canada. There is evidence that uniqueness can be explained by the geographic area's population size [40]. For example, if Montreal postal codes were uncharacteristically small compared to other cities, then one would have concerns that our uniqueness estimates would be exaggerated for other cities. However, a comparison among the Montreal postal code population sizes and those of Ottawa and Toronto is shown in Table 1. As can be seen, Montreal areas tend to be the same size or larger than the other cities. This suggests that our results are likely conservative and that uniqueness may be slightly higher in other urban areas.

**Table 1:** A comparison of postal code population sizes among Montreal, Ottawa, and Toronto.

	Ottawa		Montreal		Toronto	
**No. Letters in Postal Code**	**Mean**	**Median**	**Mean**	**Median**	**Mean**	**Median**

**1**	362,211	362,211	1,870,336	1,870,336	699,936	699,936

**2**	181,106	181,106	267,191	273,240	116,656	88,401

**3**	16,464	16,368	19,792	19,602	12,498	10,892

**4**	3,853	4,016	5,616	4,954	3,910	2,286

**5**	289	220	351	264	324	228

**6**	40	23	48	30	51	23

#### 4.4.2 Generalization to Rural Areas

**Figure 4:**
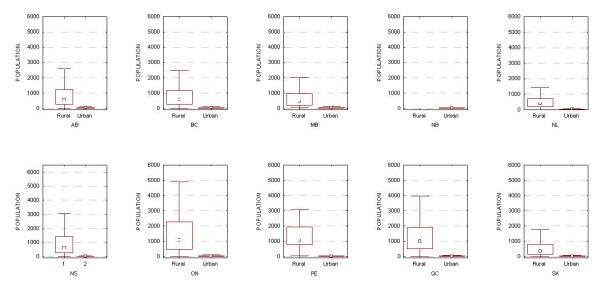
**Distribution of population sizes in postal codes for all ten provinces categorized by urban vs. rural**.

Another concern is whether our results can be generalized to rural areas in Canada. As shown in Figure 4, rural postal codes tend to have larger populations than urban ones (with the exception of New Brunswick, but in that province all postal codes are classified as urban). We used the Canada Post and the Canadian Medical Association definitions of a rural postal code, which is only one of multiple possible definitions [56], but a common one. The larger population sizes in rural postal codes means that uniqueness values will tend to be smaller than the ones presented here.

Below we provide a more direct analysis demonstrating that the uniqueness results for urban areas cannot be generalized to rural areas. This analysis is limited to the province of Ontario. We assume that these results would be generally representative to other areas of Canada.

We follow a method used by Sweeney in her estimate of population uniques in the US [39]. In her study she only had ZIP codes and the number of people within each age group living in that ZIP code, and her objective was to compute uniqueness. She computed the population uniqueness on simple demographics by randomly distributing the population living within a geographic area to the different "day of birth" bins. For instance, within a year there are 365 days and there may be 400 people living in that area who are 35 years old. She then distributed the 400 35-year-olds uniformly across all days of their birth year to obtain an actual date of birth.

First we need to ascertain whether uniqueness on the basic demographics is stable if we uniformly distribute individual birthdays across the year as we described above. We took the birth registry for Ontario for the five years from 2004 to 2008, and randomly assigned each newborn a new day of birth within their actual year of birth using a uniform distribution. For example, an Ontarian born on 1^st ^January 2005 may be reassigned to 12^th ^March 2005. We then computed uniqueness on the new day of birth and actual postal code, and compared that to uniqueness using the original day of birth.

The results are shown in Table 2. This indicates that the uniqueness on actual date of birth and maternal postal code is high and similar to our main results. This provides independent confirmatory evidence. Also, Table 2 shows that the uniform distribution of births across the days of the year results in very similar uniqueness values. The uniform distribution increases uniqueness by 3%-4% only.

**Table 2:** The difference in between real uniqueness and uniqueness estimated making the uniform distribution assumption.

		% Uniques	
**Fiscal Yr**	**Total of Births**	**Uniform Dist**.	**Real**

2004	113,220	97.8%	93.9%

2005	120,803	97.5%	94.0%

2006	125,724	97.6%	94.0%

2007	136,980	98.0%	94.5%

2008	139,278	98.2%	94.7%

Therefore, if we randomly distribute Ontarians with a known age to a particular day of birth, the impact on uniqueness is small. Following the uniform distribution strategy would give us a reasonably accurate measure of uniqueness for different postal codes.

Next, we obtained the latest information on the population sizes by age and gender from the Ontario Ministry of Finance based on the 2006 Canadian census. We also obtained the population sizes for each postal code in Ontario. For individuals living in each postal code we randomly assigned them a day of birth. Therefore, we ended up with a full date of birth and gender for each individual living in each postal code. Using those three values, we computed uniqueness on the basic demographics for each postal code. The random assignment was repeated 1000 times and the mean uniqueness taken to represent the uniqueness result for that postal code.

We then divided the postal codes into those that were rural and urban.

The median uniqueness for urban postal codes was 100% on the full date of birth and postal code only. For rural areas it was 98.1%. On the month and year of birth and postal code, 98.7% of urban Ontarians were unique, whereas only 57% of rural Ontarians were unique. Finally, only 1% of rural Ontarians are unique, whereas 85% of urban Ontarians are unique on year of birth and postal codes.

These results make clear that the high uniqueness values for urban populations will not necessarily hold for rural populations. Ontario rural postal codes tend to be larger than urban ones (median population size 1086 vs. 25), hence explaining why uniqueness drops off quite rapidly as the date of birth is generalized.

If one were to apply the policy alternatives under the 5% and 20% thresholds that we present in additional file 2 for rural areas, the policies will still be correct. Albeit, they will be conservative policies for rural areas.

#### 4.4.3 Comparison to US Results

Previous studies conducted in the US showed that the percentage of uniques on basic demographics (i.e., date of birth, ZIP code, and gender) range from 63% to 87% [39,41], the values are somewhat lower than our findings. This can be explained by the fact that US ZIP codes tend to be larger than Canadian postal codes. For example, the median population for 5-digit ZIP codes is 2,696 (the mean is 8,846), whereas in Canada the median population size for a postal code is 19 and the mean is 41. Therefore, one would expect lower uniqueness values for geographic areas with larger populations.

### 4.5 Relationship to Previous Work

It has been demonstrated that the trail of locations of hospitals that a patient has visited can be used to re-identify their records because such visit trails tend be unique or sufficiently rare [57,58]. However, hospital visit data is not part of basic demographics, whereas individual residence locations are. There is also evidence that uniqueness can be high when home and work locations of individuals are known [59], and that home address (and hence identity) can be determined by tracking the locations that individuals pass through or stop at while driving [60].

There have been no studies examining re-identification risk from residence trails, and their combination with basic demographic information.

### 4.6 Expansion to Other Quasi-identifiers

Our analysis was based on only three quasi-identifiers. These are common in many data sets used and disclosed for health research purposes. Additional quasi-identifiers, such as socio-economic variables, are also often used. The addition of these quasi-identifiers would raise uniqueness. This means that if uniqueness is high with our three quasi-identifiers then it will also be high with the addition of other socio-economic quasi-identifiers, and if it was low in our study then it may increase and exceed the thresholds if more quasi-identifiers are added.

For longitudinal data sets, some additional quasi-identifiers will not be affected by time, such as race and ethnicity. Others, such as income and language spoken at home, may vary over time. Therefore, future work which expands on our core data set should consider time variability of variables.

### 4.7 Future Work

Extensions of this work should address the data quality questions that we have raised. In particular, examination of causes of missing or inconsistent data would be needed to determine the impact on uniqueness. It is also expected that over time data quality will improve because of better data validation at the point of collection and better cross-validation with other data sources. This suggests that a repetition of this analysis in the future would provide more accurate estimates of uniqueness.

One advantage that we had was access to a population registry with long term data that we could sample from. The replication of this study in other jurisdictions without a population registry may limit the generalizability of these replications.

In other jurisdictions the cropping of codes representing areal units may not be appropriate. In our context a cropped postal code will represent an area that is a superset of the uncropped area. However, this same relationship may not hold everywhere.

### 4.8 Limitations

The postal code data we have represent where individuals were living at the beginning of the year. This does not capture any movements within a year that may occur. The impact of this effect is that our results are conservative, and may underestimate overall uniqueness. Although it is not expected that many people would be making multiple moves within a single year.

## 5 Conclusions

It is important to maintain the Canadian public's trust about the confidentiality of their health information when used for health research purposes. One way to achieve this is to de-identify the information used for research. However, there has been a dearth of studies examining what information makes Canadians identifiable. In this study we examine the re-identification risk from longitudinal basic demographics: age, gender, and postal codes over an 11 year period. We found that indeed the basic demographics can make Canadians easily re-identifiable. This risk increases as individuals' residence trails increase in duration. In this paper we provide detailed data disclosure policy options for information custodians under alternative risk thresholds.

## 6 Competing interests

The authors declare that they have no competing interests.

## 7 Authors' contributions

KEE designed the study, directed the data analysis, performed data analysis, and contributed to writing the paper. DB designed the study, directed the data analysis, and contributed to writing the paper. RT designed the study, directed the data analysis, and contributed to writing the paper. AN performed data analysis. EJ contributed to writing the paper. AV prepared the data set for analysis. All of the authors have read and approved the final manuscript.

## Pre-publication history

The pre-publication history for this paper can be accessed here:

http://www.biomedcentral.com/1472-6947/11/46/prepub

## Supplementary Material

Additional file 1 **Interactive results graphs**. This file contains interactive graphs showing the results for all years from 2 to 11, and also separately for adults and youth. Click here for file

Additional file 2 **Disclosure policies**. This file presents all of the acceptable disclosures of demographics under the 5% and 20% uniqueness thresholds. Click here for file
