# Supplementary material for: The re-identification risk of Canadians from longitudinal demographics
Source: BMC Med Inform Decis Mak. 2011 Jun 22;11:46. doi: 10.1186/1472-6947-11-46 (PMC3151203; doi:10.1186/1472-6947-11-46)
Supplement: Additional file 1 — Interactive results graphs. This file contains interactive graphs showing the results for all years from 2 to 11, and also separately for adults and youth. [file 1472-6947-11-46-S1.PDF]

Appendix A: Interactive Results Graphs

The following pages include interactive results graphs which allow you to select the level of granulatiity of the demographics and the number of years in the residence trail. The overall results, as well as those for adults only and youth only, are shown.

Please note that these are Flash objects embedded within the document, and you may get a security warning about playing embedded flash objects. You will need to allow the flash objects to play before you can see the graphs (otherwise they will just appear as blank pages). You may need to click on the center of the page with the graph to activate it.

	**The following page has an interactive graph for all individuals in our data set. This is an interactive version of the same results shown in the main body of the paper.**	

	**The following page has an interactive graph for adults in our data set. An adult is defined as ≥20 years.**	

	**The following page has an interactive graph for all youth in our data set. Youth are defined as those <20 years old.**	
