# Supplementary material for: The re-identification risk of Canadians from longitudinal demographics
Source: BMC Med Inform Decis Mak. 2011 Jun 22;11:46. doi: 10.1186/1472-6947-11-46 (PMC3151203; doi:10.1186/1472-6947-11-46)
Supplement: Additional file 2 — Disclosure policies. This file presents all of the acceptable disclosures of demographics under the 5% and 20% uniqueness thresholds. [file 1472-6947-11-46-S2.PDF]

Appendix B: Disclosure Policies

In this appendix we identify the different data disclosure policies that would be acceptable under 5% and 20% uniqueness thresholds for each number of years in a residence trail.

The values in the two right hand columns describes the percentage of the population that is unique. For example, for 1 year of data the “dd/mm/yyyy”, sex=”Y”, and 1 character of the postal code only 0.23% of the population is unique, which is below both thresholds. Therefore, the population uniqueness is shown across both thresholds.

On the other hand if we have 1 year of data, “dd/mm/yyyy” date of birth, sex=”Y”, and two characters in the postal code it would not be possible to disclose the data under the <5% threshold because 14.97% of the population is unqiue. In this case a dash is inserted in the <5% column. However, it would be possible to disclose the data under the 20% threshold.

If the percentage of unique individuals is higher than both the 5% and 20% thresholds then we do not show that in the tables below. The tables only show possible disclosure policies that would be acceptable under either or both thresholds.

	Date of Birth	Sex	Postal Code (# of digits)	Unique %	
				≤5%	≤20%
1 Year	dd/mm/yyyy	Y	1	0.23	
	dd/mm/yyyy	Y	2	-	14.97
	dd/mm/yyyy	N	1	0.14	
	dd/mm/yyyy	N	2	3.59	
	mm/yyyy	Y	1	0.02	
	mm/yyyy	Y	2	0.029	
	mm/yyyy	Y	3	2.69	
	mm/yyyy	N	1	0	
	mm/yyyy	N	2	0.02	
	mm/yyyy	N	3	0.52	
	mm/yyyy	N	4	-	8.48
	yyyy	Y	1	0	
	yyyy	Y	2	0.004	
	yyyy	Y	3	0.03	
	yyyy	Y	4	0.15	
	yyyy	N	1	0	
	yyyy	N	2	0.002	
	yyyy	N	3	0.02	
	yyyy	N	4	0.06	
	yyyy	N	5	-	13.68
		Y	1	0	
		Y	2	0	
		Y	3	0	
		Y	4	0	
		Y	5	0	
		Y	6	0	
		N	1	0	
		N	2	0	
		N	3	0	
		N	4	0	
		N	5	0	
		N	6	0	

	Date of Birth	Sex	Postal Code (# of digits)	Unique %	
				≤5%	≤20%
2 Years	dd/mm/yyyy	Y	1	1.13	
	dd/mm/yyyy	N	1	0.96	
	dd/mm/yyyy	N	2	-	12.45
	mm/yyyy	Y	1	0.05	
	mm/yyyy	Y	2	3.49	
	mm/yyyy	Y	3	-	17.94
	mm/yyyy	N	1	0.02	
	mm/yyyy	N	2	1.95	
	mm/yyyy	N	3	-	12.89
	yyyy	Y	1	0.002	
	yyyy	Y	2	0.18	
	yyyy	Y	3	-	13.1
	yyyy	Y	4	-	16.9
	yyyy	N	1	0	
	yyyy	N	2	0.06	
	yyyy	N	3	-	10.59
	yyyy	N	4	-	17.85
		Y	1	0	
		Y	2	0	
		Y	3	0.54	
		Y	4	4.82	
		N	1	0	
		N	2	0	
		N	3	0.18	
		N	4	2.75	
		N	5	-	17.40
		N	6	-	17.40

	Date of Birth	Sex	Postal Code (# of digits)	Unique %	
				≤5%	≤20%
3 Years	dd/mm/yyyy	Y	1	2.29	
	dd/mm/yyyy	N	1	2.22	
	dd/mm/yyyy	N	2	-	18.16
	mm/yyyy	Y	1	0.13	
	mm/yyyy	Y	2	-	8.7
	mm/yyyy	N	1	0.05	
	mm/yyyy	N	2	-	5.69
	yyyy	Y	1	0.006	
	yyyy	Y	2	1.23	
	yyyy	N	1	0.003	
	yyyy	N	2	0.69	
		Y	1	0	
		Y	2	0.03	
		Y	3	4.34	
		Y	4	-	13.13
		N	1	0	
		N	2	0.01	
		N	3	2.85	
		N	4	-	9.29
4 Year Years	dd/mm/yyyy	Y	1	3.33	
	dd/mm/yyyy	N	1	3.56	
	mm/yyyy	Y	1	0.29	
	mm/yyyy	Y	2	-	13.89
	mm/yyyy	N	1	0.11	
	mm/yyyy	N	2	-	10.18
	yyyy	Y	1	0.016	
	yyyy	Y	2	2.9	
	yyyy	N	1	0.009	
	yyyy	N	2	1.76	
		Y	1	0	
		Y	2	0.23	
		Y	3	-	8.34
		N	1	0	
		N	2	0.12	
		N	3	-	6.15
		N	4	-	14.87

	Date of Birth	Sex	Postal Code (# of digits)	Unique %	
				≤5%	≤20%
5 Years	dd/mm/yyyy	Y	1	3.98	
	dd/mm/yyyy	N	1	4.65	
	mm/yyyy	Y	1	0.54	
	mm/yyyy	Y	2	-	17.76
	mm/yyyy	N	1	0.22	
	mm/yyyy	N	2	-	13.94
	yyyy	Y	1	0.036	
	yyyy	Y	2	4.88	
	yyyy	N	1	0.02	
	yyyy	N	2	3.14	
		Y	1	0	
		Y	2	0.65	
		Y	3	-	12.74
		N	1	0	
		N	2	0.39	
		N	3	-	9.65
		N	4	-	19.6
6 Years	dd/mm/yyyy	Y	1	4.77	
	dd/mm/yyyy	N	1	-	5.71
	mm/yyyy	Y	1	0.91	
	mm/yyyy	N	1	0.37	
	mm/yyyy	N	2	-	17.13
	yyyy	Y	1	0.07	
	yyyy	Y	2	-	6.82
	yyyy	N	1	0.04	
	yyyy	N	2	4.63	
		Y	1	0	
		Y	2	1.23	
		Y	3	-	16.13
		N	1	0	
		N	2	0.81	
		N	3	-	12.29

	Date of Birth	Sex	Postal Code (# of digits)	Unique %	
				≤5%	≤20%
7 Years	dd/mm/yyyy	Y	1	-	5.28
	dd/mm/yyyy	N	1	-	6.61
	mm/yyyy	Y	1	1.37	
	mm/yyyy	N	1	0.58	
	yyyy	Y	1	0.1	
	yyyy	Y	2	-	8.84
	yyyy	N	1	0.08	
	yyyy	N	2	-	6.06
		Y	1	0	
		Y	2	1.92	
		Y	3	-	19.08
		N	1	0	
		N	2	1.36	
		N	3	-	14.72
8 Years	dd/mm/yyyy	Y	1	-	5.38
	dd/mm/yyyy	N	1	-	6.65
	mm/yyyy	Y	1	1.38	
	mm/yyyy	N	1	0.59	
	yyyy	Y	1	0.1	
	yyyy	Y	2	-	8.97
	yyyy	N	1	0.08	
	yyyy	N	2	-	6.24
		Y	1	0	
		Y	2	1.94	
		Y	3	-	19.13
		N	1	0	
		N	2	1.36	
		N	3	-	14.72

	Date of Birth	Sex	Postal Code (# of digits)	Unique %	
				≤5%	≤20%
9 Years	dd/mm/yyyy	Y	1	-	5.79
	dd/mm/yyyy	N	1	-	7.32
	mm/yyyy	Y	1	1.89	
	mm/yyyy	N	1	0.82	
	yyyy	Y	1	0.15	
	yyyy	Y	2	-	10.89
	yyyy	N	1	0.11	
	yyyy	N	2	-	7.62
		Y	1	0.0022	
		Y	2	2.54	
		N	1	0	
		N	2	1.87	
		N	3	-	16.88
10 Years	dd/mm/yyyy	Y	1	-	6.20
	dd/mm/yyyy	N	1	-	8.05
	mm/yyyy	Y	1	2.46	
	mm/yyyy	N	1	1.12	
	yyyy	Y	1	0.2	
	yyyy	Y	2	-	12.75
	yyyy	N	1	0.15	
	yyyy	N	2	-	9.04
		Y	1	0.005	
		Y	2	3.23	
		N	1	0	
		N	2	2.45	
		N	3	-	18.79
11 Years	dd/mm/yyyy	Y	1	-	6.23
	dd/mm/yyyy	N	1	-	8.08
	mm/yyyy	Y	1	2.5	
	mm/yyyy	N	1	1.13	
	yyyy	Y	1	0.2	
	yyyy	Y	2	-	12.98
	yyyy	N	1	0.16	
	yyyy	N	2	-	9.19
		Y	1	0.004	
		Y	2	3.24	
		N	1	0	
		N	2	2.45	
		N	3	-	18.92
